# Case Report: Cerebral metastasis following standard therapy in BRAF/KRAS wild-type colorectal cancer: an unusual presentation of disease progression

**DOI:** 10.3389/fonc.2026.1772072

**Published:** 2026-04-24

**Authors:** Nazmin Ahmed, Vishal K. Chavda, Mohammad Nazrul Hossain

**Affiliations:** 1 Ibrahim Cardiac Hospital and Research Institute, Dhaka, Bangladesh; 2 Department of Neurology, Neurosurgery and Neurogenetics; Department of Clinical and Translational Research, ChromoMed Institute, Santo Domingo, Dominican Republic

**Keywords:** brain metastasis, chemotherapy, colorectal cancer, KRAS/BRAF wild-type, TP53 mutation

## Abstract

Brain metastasis from colorectal cancer (CRC) is typically associated with advanced systemic disease and aggressive molecular features. CRC with wild-type KRAS/BRAF, microsatellite stability, and low tumor mutational burden is generally considered to have a low risk of central nervous system (CNS) dissemination. We report the case of a 66-year-old woman with sigmoid colon adenocarcinoma who initially presented with locoregionally advanced disease and distant metastasis. She received neoadjuvant chemotherapy with six cycles of mFOLFOX regimen, followed by high anterior pelvic resection with covering ileostomy and then another six cycles of adjuvant chemotherapy, achieving apparent systemic remission on serial positron emission tomography/computed tomography (PET/CT) imaging. Molecular profiling revealed a microsatellite-stable tumor with low tumor mutational burden, wild-type KRAS/NRAS/BRAF, and a TP53 mutation. The patient developed delayed multiple brain metastasis more than 1 year after completion of systemic therapy with heterogeneously enhancing intracranial lesions with surrounding vasogenic edema. She underwent neuronavigation-guided craniotomy with near-total resection of the dominant lesion, and histopathology confirmed metastatic adenocarcinoma of colorectal origin. Overall, she had an unusual pattern of disease progression in molecularly low-risk CRC, indicating that delayed CNS metastasis can occur despite apparent systemic remission. These findings challenge current assumptions regarding CNS risk stratification in CRC and raise important questions about the need for individualized neurological surveillance strategies in selected patient subgroups.

## Introduction

Brain metastasis (BM) from colorectal cancer (CRC) is uncommon, occurring in approximately 1%–4% of cases, and typically appears in patients with advanced systemic disease or aggressive molecular profiles such as KRAS or BRAF mutations (1, 2). BM usually develops late in the disease course and is often preceded by pulmonary metastasis, reflecting typical hematogenous spread patterns. CRC with wild-type KRAS and BRAF, MSI stability, and low tumor mutational burden (TMB) is generally considered to have a lower risk of central nervous system (CNS) dissemination (3, 4).

We present a rare case of advanced colon adenocarcinoma (MSI-stable, TP53-mutated, and KRAS/BRAF-negative) that developed multiple cerebral metastases following apparently successful surgery and chemotherapy. This case illustrates an atypical and clinically important pattern of disease progression and challenges the prevailing assumption that molecularly favorable CRC is at low risk for CNS dissemination and raises questions regarding surveillance strategies in selected subgroups.

## Case description

A 66-year-old woman with a history of type 2 diabetes mellitus (DM) and hypothyroidism presented with intermittent hematochezia for 3 months, along with generalized weakness, loss of appetite, and unintentional weight loss for 6 months. Her family history was significant for breast cancer in her mother and CRC in a maternal uncle and aunt. She was evaluated by a general surgeon and diagnosed with carcinoma of the sigmoid colon following colonoscopy-guided biopsy on 30 December 2023. Colonoscopy revealed an ulceroproliferative lesion extending from 23 to 30 cm from the anal verge. Histopathological examination confirmed moderately differentiated adenocarcinoma.

For further evaluation and staging, contrast-enhanced computed tomography (CECT) of the whole abdomen performed on 9 January 2024 demonstrated circumferential wall thickening of the sigmoid colon with a contrast enhancing mass measuring 7.7 × 9.6 × 10.2 cm, eroding the sigmoid wall and communicating with the colonic lumen, with central necrosis. Enlarged multiple para-aortic lymph nodes were noted, the largest measuring 1.7 cm in short-axis diameter. Furthermore, there were multiple enlarged lymph nodes near the left adrenal gland, and perihepatic fluid collection with enhancing lung nodules was noted in both lung fields, suggestive of metastasis. TNM staging was T4aN2bM1. Serum tumor markers showed elevated CA-125 (175 U/mL on 6 January 2024), while CA-19-9 (4.53 U/mL, 6 January 2024) and carcinoembryonic antigen (CEA) (1.39 ng/mL, 11 January 2024) were within normal limits. For downstaging of the tumor and facilitating surgical resection, the patient received six cycles of neoadjuvant chemotherapy (NACT) with mFOLFOX (5-fluorouracil, leucovorin, and oxaliplatin), started from 14 January 2024. Cycle 6 was received on 2 March 2024 and the patient was again re-evaluated with CECT scan of the chest and abdomen on 24 April 2024, which revealed perforation in the sigmoid colon and matted small bowel loops in the left side of the pelvic cavity communicating with extensive irregular pelvic collection with air locules, suggestive of pelvic abscess. There was metastatic nodules in the right lung, reduced in number, size, and distribution with partial collapse of D12 vertebrae. She then underwent high anterior resection with covering ileostomy on 24 April 2024.

**Figure 1:**
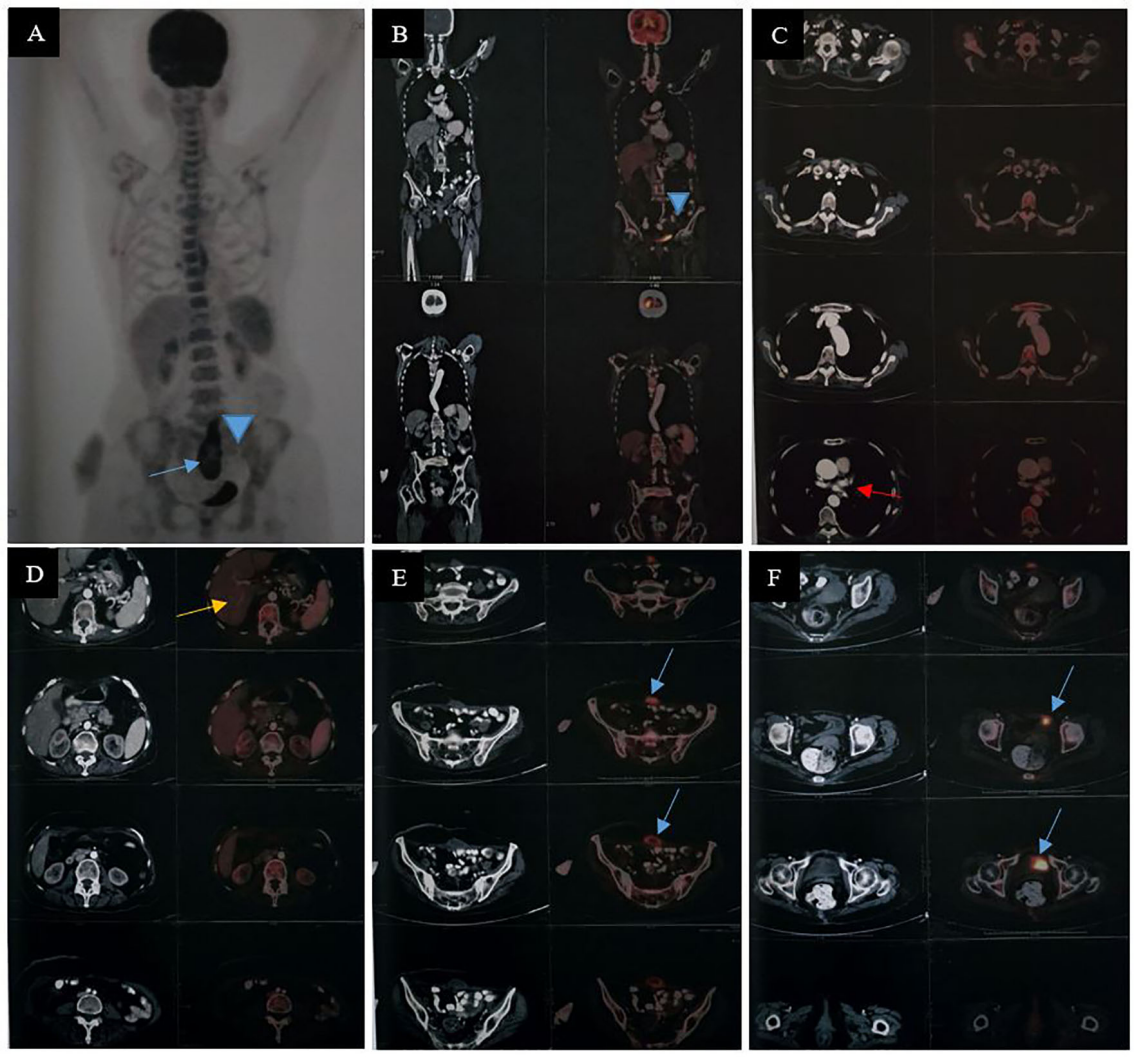
Whole body PET/CT, maximum intensity projection (MIP) (17 October 2024) demonstrated no abnormal FDG uptake in the anastomosis site [**(A, B)** marked by a blue arrowhead]. There was a marginal FDG uptake area (SUV max: 6.2) with non-avid central component noted in the anterior lower abdominal wall (7 × 4.1 × 9.8 cm) in the midline following the incision line, likely inflammatory collection [**(A, E, F)** marked by a blue arrow]. Benign left lung nodule [**(C)** marked by red arrow] with mild hepatomegaly noted [**(D)** marked by an orange arrow].

**Figure 2:**
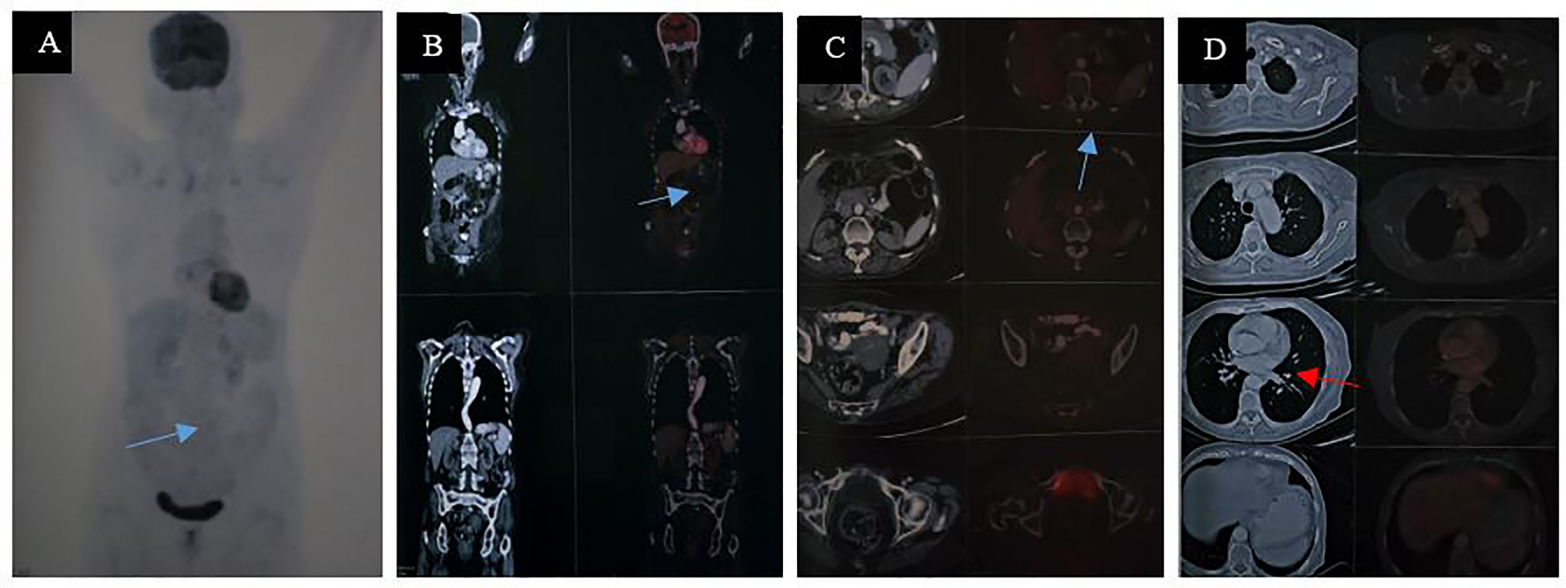
Repeat PET/CT, maximum intensity projection (MIP) on 16 January 2025 was in favor of the resolution of a previous FDG-avid abdominal wall focus [**(A–C)** the site was marked by a blue arrow] and the lung nodules [**(D)** marked by a red arrow].

Because of the development of a fecal fistula, surgical toileting with drain fixation was performed on 27 May 2024. Follow-up CT scan of the chest and abdomen on 8 July 2024 demonstrated successful colo-colic anastomosis, with no evidence of abdominopelvic collection or definite abdominal lymphadenopathy. The patient then resumed adjuvant systemic chemotherapy with mFOLFOX starting on 11 July 2024, completing cycle 12 on 24 September 2024. On 17 October 2024, positron emission tomography/computed tomography (PET/CT) scan of the whole body demonstrated no abnormal FDG uptake in the anastomosis site. There was a marginal FDG uptake area (SUV max: 6.2) with non-avid central component noted in the anterior lower abdominal wall (7 × 4.1 × 9.8 cm) in the midline following the incision line, likely inflammatory collection, non-avid left lung nodule with mild hepatomegaly (**Figure 1**). As PET/CT was in favor of excellent disease control with no FDG avid areas in the abdomen, suggestive of recurrence/residual of the lesion, no radiotherapy was considered. Patient was followed up and she underwent ileostomy reversal with excision of sinus tract on 9 November 2024. Repeat PET/CT on 16 January 2025 was in favor of resolution of the lung findings and previous FDG-avid abdominal wall focus (**Figure 2**).

**Figure 3:**
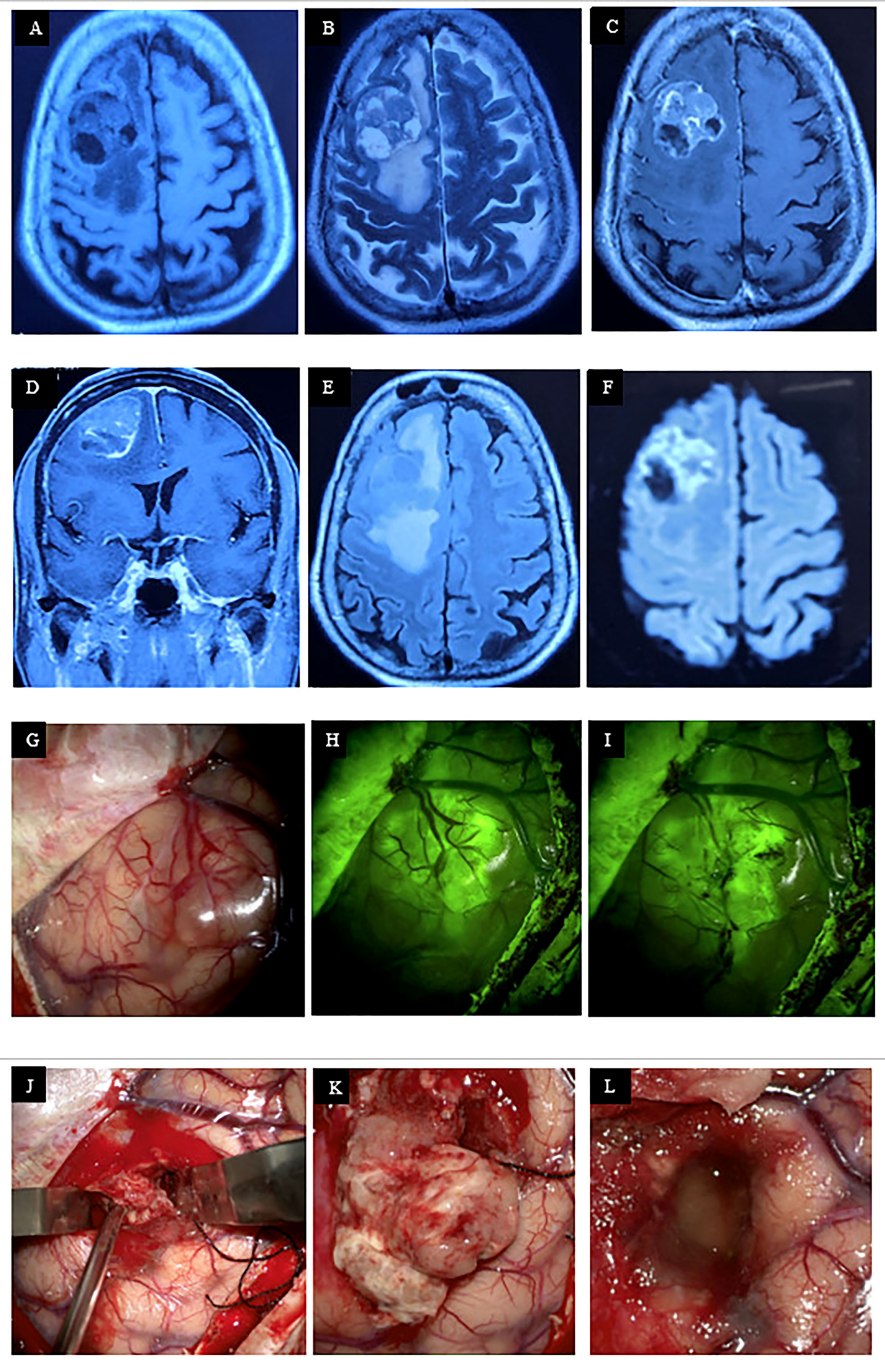
MRI of brain showing a well-defined 3.6 × 3.3 cm, heterogeneously hypointense lesion in the right prefrontal cortex **(A)**, which becomes iso-hyperintense in T2WI, devoid of intrinsic or marginal flow voids **(B)**. After administration of gadolinium, there is inhomogeneous contrast enhancement with apparent dural involvement **(C, D)**. There is moderate vasogenic edema **(E)** with partial diffusion restriction **(F)**. Intraoperative photograph demonstrates sequential steps of tumor resection, started with durotomy and microscopic visualization of the tumor **(G)**, fluorescein enhancement **(H)**, surface devascularization **(I)**, removal of the solid part, following the cleavage plane **(J)**. The solid part was whitish gray, partly soft and partly firm with moderate vascularity **(K)**. Hemostasis achieved with fibrillary **(L)**.

On 30 January 2025, a molecular study of colonic cancer detected TP53 mutation, MSI-stable, TMB-low (4 mut/Mb), HER2 negative, and wild-type KRAS/NRAS/BRAF. These findings indicated a molecularly favorable phenotype with no high-risk CRC mutations for CNS spread. Patients had a favorable disease course till 30 October 2025 when repeat PET/CT scan demonstrated newly developed hypermetabolic (SUVmax: 6.7), enhancing intracranial space-occupying lesion (ICSOL) involving the right frontal lobe (31.5 × 24.4 mm) anteriorly with perilesional edema, likely BM. There was a newly developed hypermetabolic nodular lesion (16 × 12 mm) on the left side of the pelvic cavity, suggestive of peritoneal seedling. For further evaluation, MRI of the brain with contrast (3 November 2025) showed multiple heterogeneous contrast enhancing lesion in right frontal (3.6 × 3.3 cm) and right temporal lobes (**Figure 3**). MRS revealed ↓NAA, ↑choline, and lactate peak consistent with metastasis. Moreover, a perfusion study was suggestive of hypervascular metastases due to elevated rCBV/rCBF. Thus, multiple intracranial metastatic lesions were confirmed.

The patient was then admitted into our department for further evaluation and definitive management of cerebral metastasis. On 19 November 2025, she underwent right sided fronto-parietal craniotomy and near total removal of the frontal SOL. With all aseptic precautions, under G/A, neuro-navigation guided tumor topography was performed and skin incision was planned accordingly. Horseshoe incision is given on right frontoparietal region, centering the tumor boundary. After subgaleal dissection, a 6 × 4 cm frontoparietal bone flap was elevated. The dura was incised in a cruciate fashion. Following dural opening, the tumor was visualized as a reddish-white area under the PENTERO 800 surgical microscope. The operative field was then examined using a YELLOW 560-nm fluorescent filter. A well-defined fluorescence-enhanced area was visible under the fluorescence filter. Tumor debulking was performed using CUSA. A partially well-defined cleavage plane was observed between the tumor and the surrounding brain parenchyma. Near-total removal of the space-occupying lesion (SOL) was achieved. Dural hitch sutures were placed, and watertight dural closure was ensured. The bone flap was fixed with titanium miniplates and screws. The wound was closed in layers, with no drain left *in situ*. Tissue samples were sent for histopathological examination and immunohistochemistry.

The patient had an uneventful postoperative recovery. She was discharged on the fifth postoperative day and referred to an oncologist for further management. Histopathology and immunohistochemistry findings were consistent with metastatic adenocarcinoma. Following completion of chemotherapy, surveillance whole-body PET/CT demonstrated no fluorodeoxyglucose (FDG)–avid extracranial disease, suggesting apparent systemic remission.

## Discussion

BMs from CRC are rare, late-occurring events that usually indicate advanced systemic disease and are associated with poor prognosis (1). In 2019, Sun and his colleagues conducted whole-exome sequencing (WES) and whole-genome sequencing (WGS) of 19 patient-matched trios consisting of BM, primary CRC, and surrounding normal tissue. Fifty-four nonsynonymous mutations in 35 (DNA damage response) DDR genes were identified in their 19 BM tissues. Among them, TP53 was the gene with the highest mutation rate (74%, 14/19) (5). Beside this, several studies suggested that most local and distant metastases originate from separate subclones within the primary tumor, highlighting the genetic divergence and heterogeneity present in metastatic diseases (5–7). An increased mutation frequency of KRAS/NRAS and PIK3CA genes has been observed in BM samples (8, 9). Following those studies, it has been postulated that BM generally associated with aggressive tumor biology, lung metastasis, TP53 mutation, BRAF/KRAS mutations, higher MSI level, or incomplete systemic control (8, 10, 11).

**Figure 4:**
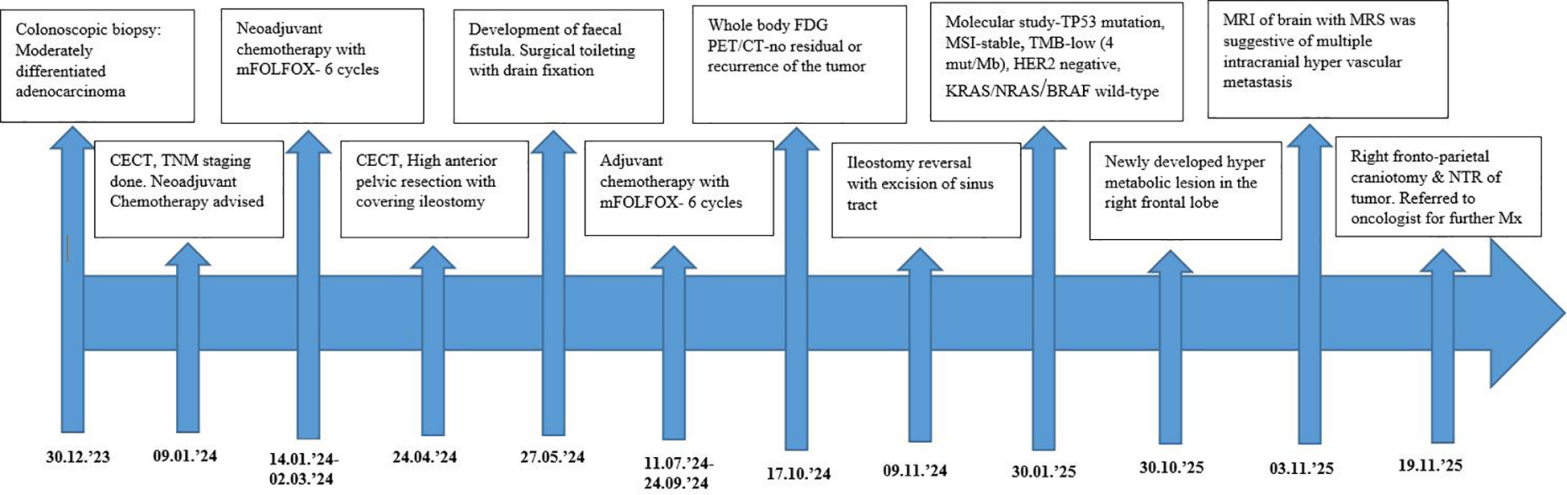
Schematic representation of the timeline, demonstrating disease course and the patient’s care.

In contrast to these typical patterns, the present case demonstrates an atypical clinical course (**Figure 4**). Despite the absence of KRAS, NRAS, and BRAF mutations, microsatellite stability, low TMB, and an initial favorable response to multimodal therapy, including surgery and 12 cycles of oxaliplatin-based chemotherapy, the patient developed delayed multiple BMs after apparent systemic remission on whole-body PET/CT. This observation challenges the prevailing assumption that molecularly “low-risk” CRC is unlikely to metastasize to the CNS and shows the limitations of relying on conventional molecular stratification for predicting metastatic behavior. Emerging evidence suggests that *TP53* mutations may drive more aggressive or unpredictable metastatic behavior despite otherwise favorable profiles (12, 13). The blood–brain barrier limits chemotherapy penetration, enabling micro-metastatic tumor cells to evade systemic treatment. Even after apparent systemic remission, selected cases of delayed cerebral metastasis have been reported in the literature (10, 12).

The premier method for identifying BMs is magnetic resonance imaging (MRI), which provides outstanding soft tissue contrast and a detailed representation of tissue structure (14). BMs from CRC can manifest as mucinous or protein-rich lesions with low T2W signal intensity. However, our reported case presented with a well-circumscribed dural-based lesion, which manifested as heterogeneously hypointense in T1WI, heterogeneously hyperintense in T2WI with inhomogeneous contrast enhancement. This dural-based well-circumscribed metastatic pattern with substantial necrotic and cystic BM represented atypical neuroimaging presentation (15). Besides this, BM spread through the bloodstream and typically develops at the junction of gray and white matter or in watershed areas, where the diameters of arterioles are smaller. Most often, the lesion is surrounded by disproportionate vasogenic edema (16). Considering these features, our reported case predominantly involved gray matter to infiltrate leptomeninges, surrounded by moderate perilesional edema.

Over the past 10 years, significant progress has been made in the treatment of CRC due to our improved understanding of the molecular pathways and the biomarkers associated with the progression of the disease (17). To achieve this, the researchers are investigating the potential of multi-gene panel testing to align patients with targeted therapies according to the molecular characteristics of their tumors. This strategy is being utilized to create customized treatment plans for each patient with CRC, integrating biomarkers such as KRAS, BRAF, MSI, and HER2 status into the clinical decision-making process (17). Alongside the previously mentioned points, the emerging application of artificial intelligence (AI) and machine learning-enhanced models marks a significant advancement in the battle against CRC. Deep learning methods can merge multiomic datasets to discover intricate biomarker signatures that conventional approaches might overlook. AI algorithms can also forecast how individuals with CRC will react to specific treatments based on their molecular biomarker profiles (18). Beside this, machine learning algorithms could predict reactions to immune checkpoint inhibitors by identifying molecular patterns, including microsatellite instability-high (MSI-H) or TMB (19).

Considering the rare and late events, little information is available in the existing literature regarding the prognosis of BM following treatment of patients with CRC. In a recent systematic review and meta-analysis, including 541,244 patients with CRC, 1,547 patients were diagnosed as having BM, whereas the median survival duration varied from 2 to 9.6 months, while the overall survival (OS) extended to as much as 41.1 months in individuals receiving a multimodal treatment approach. Various factors, such as age, serum levels of CEA, the presence of multiple metastatic sites, the quantity of brain lesions, and the existence of the KRAS mutation, were indicators of OS (11). However, cases such as this raise important questions regarding whether specific molecular or clinical subgroups, such as patients with TP53 mutations, strong family history, or prolonged survival following effective systemic therapy may warrant closer neurological monitoring.

Limitation of the study: This is a single case report observation of the delayed cerebral metastasis of BRAF/CRAS wild-type CRC. Further research is needed to clarify risk factors for delayed CNS metastasis and to determine whether surveillance strategies could improve early detection and outcomes in selected patients.

Strength of the study: This case highlights the following:

1. CNS metastasis can occur in molecularly low-risk CRC.
2. Surveillance guidelines may need refinement for certain molecular subgroups.
3. The presence of TP53 mutation and strong family history may contribute to a more aggressive phenotype despite KRAS/BRAF negativity.
4. Atypical imaging features sometimes mislead the diagnosis.

## Conclusion

This case demonstrates that patients with CRC with BRAF- and KRAS-negative tumors and initially good systemic response can still develop delayed BMs. Clinicians should maintain a high index of suspicion for neurological symptoms during follow-up. Further research is required to determine whether specific molecular or clinical subgroups might benefit from targeted CNS surveillance.

## Patient perspective

The patient and her family expressed concern that the BMs appeared despite successful surgeries and chemotherapy. They emphasized the importance of monitoring neurological symptoms early.

## Data Availability

The original contributions presented in the study are included in the article/supplementary material. Further inquiries can be directed to the corresponding author.

## References

[1] Mjahed RB Astaras C Roth A Koessler T . Where are we now and where might we be headed in understanding and managing brain metastases in colorectal cancer patients? Curr Treat Options Oncol. (2022) 23:980–1000. doi: 10.1007/s11864-022-00982-0. PMID: 35482170 PMC9174111

[2] Sarfraz Z Jayram D Ozair A Hodgson L Bellur S Maharaj A . Survival in patients with colorectal cancer and isolated brain metastases: Temporal trends and prognostic factors from the National Cancer Database (2010–2020). Cancers (Basel). (2025) 17:2531. doi: 10.3390/cancers17152531. PMID: 40805227 PMC12345898

[3] Hammoud Z Popoff A Potti C Nasser H . Metastatic disease of the lung. In: Cancer metastasis through the lymphovascular system. Springer International Publishing, Cham (2022). p. 447–56. doi: 10.1007/978-3-030-93084-4_41

[4] Santos FA Reis RM Barroti LC Pereira AAL Matsushita MM de Carvalho AC . Overall survival, BRAF, RAS, and MSI status in patients who underwent cetuximab after refractory chemotherapy for metastatic colorectal cancer. J Gastrointest Cancer. (2024) 55:344–54. doi: 10.1007/s12029-023-00964-x. PMID: 37608030

[5] Sun J Wang C Zhang Y Xu L Fang W Zhu Y . Genomic signatures reveal DNA damage response deficiency in colorectal cancer brain metastases. Nat Commun. (2019) 10:3190. doi: 10.1038/s41467-019-10987-3. PMID: 31320627 PMC6639368

[6] Giannakis M Mu XJ Shukla SA Qian ZR Cohen O Nishihara R . Genomic correlates of immune-cell infiltrates in colorectal carcinoma. Cell Rep. (2016) 15:857–65. doi: 10.1016/j.celrep.2016.03.075. PMID: 27149842 PMC4850357

[7] Naxerova K Reiter JG Brachtel E Lennerz JK van de Wetering M Rowan A . Origins of lymphatic and distant metastases in human colorectal cancer. Sci (1979). (2017) 357:55–60. doi: 10.1126/science.aai8515. PMID: 28684519 PMC5536201

[8] Medici B Benatti S Dominici M Gelsomino F . New frontiers of biomarkers in metastatic colorectal cancer: Potential and critical issues. Int J Mol Sci. (2025) 26:5268. doi: 10.3390/ijms26115268. PMID: 40508077 PMC12154287

[9] Tie J Lipton L Desai J Gibbs P Jorissen RN Christie M . KRAS mutation is associated with lung metastasis in patients with curatively resected colorectal cancer. Clin Cancer Res. (2011) 17:1122–30. doi: 10.1158/1078-0432.CCR-10-1720. PMID: 21239505

[10] Ruzzo A Graziano F Palladino S Fischer NW Catalano V Giordani P . Clinical impact of TP53 functional mutations in patients with metastatic colorectal cancer treated with bevacizumab and chemotherapy. Oncologist. (2025) 30(3):oyae277. doi: 10.1093/oncolo/oyae277. PMID: 39436921 PMC11954512

[11] Müller S Köhler F Hendricks A Kastner C Börner K Diers J . Brain metastases from colorectal cancer: A systematic review of the literature and meta-analysis to establish a guideline for daily treatment. Cancers (Basel). (2021) 13:900. doi: 10.3390/cancers13040900. PMID: 33669974 PMC7924831

[12] Russo A Bazan V Iacopetta B Kerr D Soussi T Gebbia N . The TP53 Colorectal Cancer International Collaborative Study on the prognostic and predictive significance of p53 mutation: Influence of tumor site, type of mutation, and adjuvant treatment. J Clin Oncol. (2005) 23:7518–28. doi: 10.1200/JCO.2005.00.471. PMID: 16172461

[13] Jung M Ahn JB Chang JH Suh CO Hong S Roh JK . Brain metastases from colorectal carcinoma: Prognostic factors and outcome. J Neuro-Oncol. (2011) 101:49–55. doi: 10.1007/s11060-010-0214-9. PMID: 20467783

[14] Brindle KM Izquierdo-García JL Lewis DY Mair RJ Wright AJ . Brain tumor imaging. J Clin Oncol. (2017) 35:2432–8. doi: 10.1200/JCO.2017.72.7636. PMID: 28640699

[15] Pope WB . Brain metastases: neuroimaging. Handb Clin Neurol. (2018) 149:89–112. doi: 10.1016/B978-0-12-811161-1.00007-4, PMID: 29307364 PMC6118134

[16] Kaufmann TJ Smits M Boxerman J Huang R Barboriak DP Weller M . Consensus recommendations for a standardized brain tumor imaging protocol for clinical trials in brain metastases. Neuro Oncol. (2020) 22:757–72. doi: 10.1093/neuonc/noaa030. PMID: 32048719 PMC7283031

[17] Yang Z Wang X Zhou H Jiang M Wang J Sui B . Molecular complexity of colorectal cancer: Pathways, biomarkers, and therapeutic strategies. Cancer Manag Res. (2024) 16:1389–403. doi: 10.2147/CMAR.S481656. PMID: 39403607 PMC11472760

[18] Yang J Huang J Han D Ma X . Artificial intelligence applications in the treatment of colorectal cancer: A narrative review. Clin Med Insights Oncol. (2024) 18:11795549231220320. doi: 10.1177/11795549231220320. PMID: 38187459 PMC10771756

[19] Gustav M Reitsam NG Carrero ZI Loeffler CML van Treeck M Yuan T . Deep learning for dual detection of microsatellite instability and POLE mutations in colorectal cancer histopathology. NPJ Precis Oncol. (2024) 8:115. doi: 10.1038/s41698-024-00592-z. PMID: 38783059 PMC11116442

